# Metabolic responses of plasma to extreme environments in overwintering Tibetan frogs *Nanorana parkeri*: a metabolome integrated analysis

**DOI:** 10.1186/s12983-021-00428-7

**Published:** 2021-08-28

**Authors:** Yonggang Niu, Xuejing Zhang, Haiying Zhang, Tisen Xu, Lifeng Zhu, Kenneth B. Storey, Qiang Chen

**Affiliations:** 1 School of Life Sciences, Dezhou University, Dezhou, 253023 Shandong China; 2 School of Life Sciences, Lanzhou University, Lanzhou, 730000 Gansu China; 3 School of Life Sciences, Nanjing Normal University, Nanjing, China; 4 Department of Biology, Carleton University, Ottawa, ON K1S 5B6 Canada

**Keywords:** Overwintering, *Nanorana parkeri*, Metabolomics, Plasma

## Abstract

Many animals lower their metabolic rate in response to low temperatures and scarcity of food in the winter in phenomena called hibernation or overwintering. Living at high altitude on the Tibetan Plateau where winters are very cold, the frog *Nanorana parkeri*, survives in one of the most hostile environments on Earth but, to date, relatively little is known about the biochemical and physiological adjustments for overwintering by this species. The present study profiled changes in plasma metabolites of *N. parkeri* between winter and summer using UHPLC-QE-MS non-target metabolomics in order to explore metabolic adaptations that support winter survival. The analysis showed that, in total, 11 metabolites accumulated and 95 were reduced in overwintering frogs compared with summer-active animals. Metabolites that increased included some that may have antioxidant functions (canthaxanthin, galactinol), act as a metabolic inhibitor (mono-ethylhexylphthalate), or accumulate as a product of anaerobic metabolism (lactate). Most other metabolites in plasma showed reduced levels in winter and were generally involved in energy metabolism including 11 amino acids (proline, isoleucine, leucine, valine, phenylalanine, tyrosine, arginine, tryptophan, methionine, threonine and histidine) and 4 carbohydrates (glucose, citrate, succinate, and malate). Pathway analysis indicated that aminoacyl-tRNA biosynthesis, phenylalanine, tyrosine and tryptophan biosynthesis, and nitrogen metabolism were potentially the most prominently altered pathways in overwintering frogs. Changes to these pathways are likely due to fasting and global metabolic depression in overwintering frogs. Concentrations of glucose and urea, commonly used as cryoprotectants by amphibians that winter on land, were significantly reduced during underwater hibernation in *N. parkeri*. In conclusion, winter survival of the high-altitude frog, *N. parkeri* was accompanied by substantial changes in metabolomic profiles and this study provides valuable information towards understanding the special adaptive mechanisms of *N. parkeri* to winter stresses.

**Supplementary Information:**

The online version contains supplementary material available at 10.1186/s12983-021-00428-7.

## Background

Amphibians in north temperate climates are generally forced to enter into a dormant state in the winter due to stresses that include low or subzero temperatures, a lack of food resources, and other associated stresses such as hypoxia or dehydration [1–3]. Two basic overwintering strategies are utilized by these species—terrestrial versus aquatic hibernation and both strategies present survival challenges [4]. Terrestrially hibernating amphibians can be exposed to desiccation due to their highly water-permeable skins and, if temperatures fall below about 0 °C, whole body freezing can be triggered. However, various species have developed effective mechanisms to survive and thrive, relying on profound tolerances of dehydration and somatic freezing [5–7]. Freeze tolerance is a striking survival strategy for dealing with subzero temperatures and has been extensively studied in multiple terrestrially hibernating amphibian species [8, 9]. However, amphibians that overwinter in an aquatic environment are not typically subjected to the above stresses, unless the water in a pond freezes right to the bottom. Once the surface of a pond freezes over, the oxygen content of the water begins to decline due to respiration among all resident organisms. As a result, amphibians must endure increasingly hypoxic conditions (sometimes even anoxia) since they can only rely on the surrounding water for gas and ion exchange across their skin. Hence, amphibians that successfully hibernate underwater face challenges of hypoxia but freezing only rarely [1]. In general, there are two paramount physiological mechanisms that allow amphibians to survive the winter underwater: one is protecting themselves against cold and/or hypoxia injury and the other is maintaining long-term metabolic homeostasis [8, 10–13].

Protective mechanisms against winter cold have been widely explored in a number of overwintering amphibians. Among freeze tolerant terrestrially hibernating species these mechanisms include accumulation of low molecular mass cryoprotectants (e.g., glucose, urea, glycerol) and production of ice-binding proteins that can trigger and regulate where ice forms [8, 13, 14]. Given that preserving membrane structure and function plays a significant role in living in extremely cold environments [15], membrane adaptation (e.g., altering membrane lipid composition) is also essential for winter survival [16]. Metabolic adjustments are also made to support long-term cold exposure including strategies to conserve endogenous fuels and minimizing energy expenditures by utilizing metabolic rate depression [17]. Moreover, animals can sustain activity by thermal compensation strategies to exploit resources from the environment [18, 19]. Metabolic depression is a widespread and important survival strategy for overwintering amphibians, that has been documented for the frogs *Rana catesbeiana* and *R. pipiens* [20, 21], the cane toad *Bufo marinus* [22], and the Andean toad *B. spinulosus* [23]. Although the specific metabolic adjustments to cold can vary among species [8], these physiological adjustments depend on a reorganization of the metabolic network as well as coordination with respect to fuel resource allocation.

Metabolomics (also known as metabonomics or metabolic profiling) is regarded as complementary to genomics, transcriptomics, and proteomics and has greatly expanded the scope for investigating changes in animal metabolism from a systems biology approach [24–26]. This approach enables global assessment of low molecular mass metabolites within cells and tissues and allows analysis of their biological significance as responses to environmental stressors [27, 28]. Metabolomic analysis provides good prospects in the search for new biomarkers of winter hardiness, that can enhance our understanding of energy balance, metabolic changes, and biological functions of endogenous metabolites [29–31]. A metabolomics approach has been used previously to analyze metabolic changes that support overwintering in several species including thirteen-lined ground squirrels (*Ictidomys tridecemlineatus*) [32–34], Syrian hamsters (*Mesocricetus auratus*) [35], common cutworms (*Spodoptera litura*) [30], and wolf spiders (*Schizocosa stridulans*) [31] but, to date, has not been applied to overwintering by amphibians or reptiles. The present study provides the first metabolomics analysis of a frog species, the Xizang plateau frog, *Nanorana parkeri* (Anura, Dicroglossidae), that lives in one of the most extreme environments on Earth, the Qinghai-Tibet Plateau.

Winter survival adaptations are well documented for various amphibian species, mainly those found in Europe and North America, and generally take two main forms: under water hibernation where hypoxia can become a problem in ice-covered ponds or terrestrial hibernation where adaptations to allow survival of whole body freezing are often necessary [1, 8, 9, 11, 12, 14]. These winter survival options have received limited attention to date among high altitude species. *N. parkeri* is a singularly useful subject for studies of high-altitude adaptation owing to its broad geographic distribution on the Qinghai Tibet Plateau, ranging from 2850 to 5100 m above sea level (a.s.l.) [36]. *N. parkeri* has been the focus of several studies investigating genomic adaptations to extreme high altitude [37–39], but our understanding of this frog’s physiological and biochemical adaptations that support hibernation remains limited. We found that most *N. parkeri* individuals overwinter in caves underwater at depths of 10–40 cm and some ponds were ice-covered. Recordings of temperature in overwintering sites typically occupied by *N. parkeri* during December showed that hibernacula temperatures were close to 0 °C and overwintering frogs suffered at subzero temperatures. Therefore, overwintering *N. parkeri* face multiple challenges from low oxygen, low temperature and even body freezing when ponds freeze completely. We have shown previously that overwintering *N. parkeri* exhibit oxidative stress as measured by oxidative damage and a significant reduction in antioxidant capacity [40]. Compared to summer-active frogs, overwintering *N. parkeri* showed a temperature-independent metabolic rate depression at multiple levels: whole animal, mitochondria, and expression of key enzymes [41]. Overwintering *N. parkeri* also show a weak freeze tolerance, that was supported by substantial changes in metabolomic profiles of liver and skeletal muscle [42].

The present study uses a metabolomics approach to provide a detailed inventory of blood metabolite levels, comparing summer and winter frogs, to seek adaptive changes in blood chemistry during natural wintering in cold-water shallow ponds as compared with summer active frogs. Additional assays assessed four small-molecule metabolites (glucose, urea, lactate, and glycerol) that have been linked with cryoprotection in other amphibians. Overall, this study provides novel information on the molecular mechanisms of overwintering in this high-altitude Xizang plateau frog and could provide key data that could aid conservation of this remote species.

## Materials and methods

### Sample collection

**Fig. 1:**
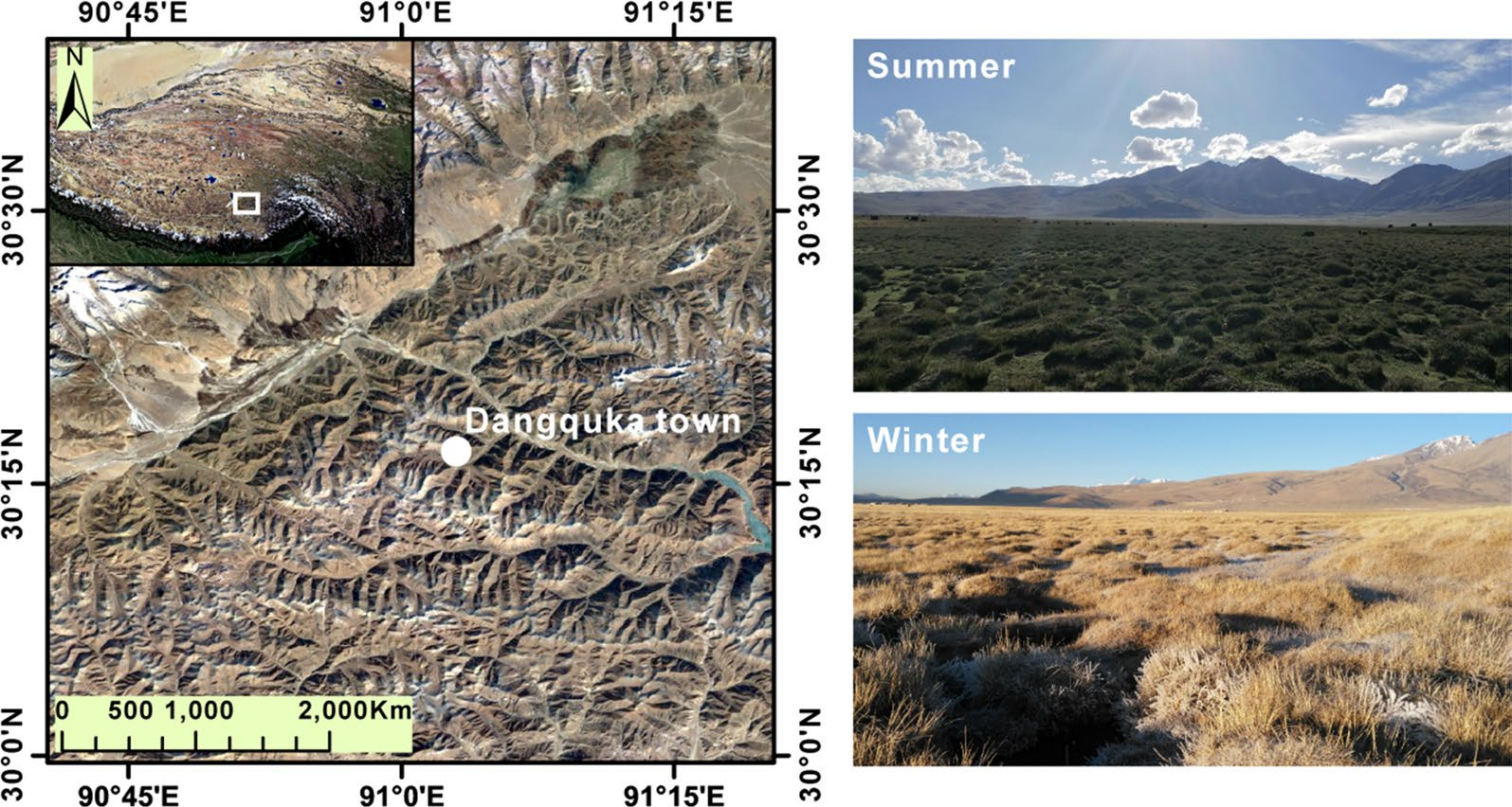
Localization of the sampling sites and habitat landscape of the frogs *Nanorana parkeri* in the summer and winter

Adult male *N. parkeri* were sampled by hand in Dangquka town (30.28° N, 91.05° E, 4280 m a.s.l.), Tibet, China (Fig. 1). Summer frogs (mid-July, 2018) were collected from marshes around the edges of ponds and winter unfrozen frogs (mid-December, 2018) were collected from the bottom of shallow ponds, respectively. At the time of sampling, the mean ambient temperature in the microhabitat was 19.32 ± 1.14 °C (n = 30) in summer and mean water temperature was 3.55 ± 0.56 °C (n = 30) in winter, respectively. Mean body mass was 4.24 ± 0.15 g (n = 11) in summer-collected frogs and 4.44 ± 0.17 g (n = 11) in winter-collected frogs. Mean snout-vent length was 4.15 ± 0.07 cm (n = 11) in summer-collected frogs and 4.13 ± 0.06 cm (n = 11) in winter-collected frogs. All animals were euthanized by decapitation near the sampling site. Approximately 200 µL of blood was drawn from each individual and added into a heparinized tube. All blood samples were immediately centrifuged at 3000*g* for 10 min and the upper layer of plasma was collected. Plasma was frozen in liquid nitrogen and stored at − 80 °C for LC-MS and biochemical analysis. Summer and winter plasma samples were shipped on dry ice to Biotree Biotech Co., Ltd. (Shanghai, China) to be analyzed by LC-MS.

### Metabolite extraction for LC-MS analysis

Plasma samples (100 µL, n = 11 for each group) were thawed at room temperature and mixed with 300 µL of methanol (containing 1 µg mL^− 1^ 2-chloro-l-phenylalanine). The mixtures were vortexed for 30 s by a vortex mixer, ultrasound-treated for 10 min in ice water with an ultrasonic apparatus (PS-60AL, Leidebang Electronics Co., Ltd., Shenzhen, China), incubated for 1 h at − 20 °C to precipitate proteins, and finally centrifuged at 13,800*g* for 15 min at 4 °C. The resulting supernatants from the samples were transferred into LC-MS vials for subsequent UHPLC-QE Orbitrap/MS analysis. Quality control (QC) samples (n = 11) were also prepared by mixing an equal aliquot (10 µL) of supernatants from all of the samples. During the instrument analysis, a QC sample was inserted every 3–4 samples to monitor the repeatability of the analysis process.

### UHPLC-QE orbitrap/MS analysis

The LC-MS-based metabolic analyses were carried out using a UHPLC system (1290, Agilent Technologies) with a UPLC HSS T3 column (2.1 mm × 100 mm, 1.8 μm, Waters) coupled to a Q Extractive™ Orbitrap Mass Spectrometer (Thermo Fisher Scientific, USA) in ESI positive (POS) and negative (NEG) ion modes. Formic acid (0.1%) and ammonium acetate (5 mM) in water were used as the mobile phase A for positive and negative ion modes, respectively. Acetonitrile was used as the mobile phase B. The injection volume was 1 µL and the flow rate was 500 µL min^− 1^ through a 12 min elution gradient (0 min, 1% B; 1 min, 1% B; 8 min, 99% B; 10 min, 99% B; 10.1 min, 1% B; 12 min, 1% B). An information-dependent acquisition (IDA) mode of the mass spectrometer was used to acquire MS/MS spectra, and the acquisition software (Xcalibur 4.0.27, Thermo) continuously evaluates the full scan survey MS data as it collects and triggers the acquisition of MS/MS spectra depending on preselected criteria. ESI source conditions were set as follows: the spray voltages were 3.8 kV (POS) and 3.1 kV (NEG), sheath gas flow rate was 45 Arb, aux gas flow rate was 15 Arb, capillary temperature was 320 °C, full ms resolution was 70,000, MS/MS resolution was 175 00, and the collision energy was 20/40/60 eV in NCE model, respectively [43].

### LC-MS data preprocessing and annotation

ProteoWizard software was used to convert the MS raw data (.raw) files to the mzML format, then retention time (RT) alignment and the identification, extraction, integration, and normalization of peaks were processed using R package XCMS (version 3.2). The data were filtered by the following criterion: only the peak area data with blank values no more than 50% in a single group or not more than 50% in all groups were retained. Outliers were filtered out according to the coefficient of variation (relative standard deviation, RSD > 30% for excluding data points). Missing values were recorded by the numerical simulation method that is to fill in half of the minimum value. A matrix was generated consisting of the sample information, retention time (RT), mass to charge ratio (m/z), and peak intensity. Then peak annotation was carried out using Compound Discover (version 2.0, Thermo) and OSI-SMMS (version 1.0, Dalian Chem Data Solution Information Technology Co. Ltd.) integrated with mzcloud database and in-house MS/MS database (Biotree Biotech Co., Ltd. Shanghai, China) [44]. The internal standard normalization method was employed for the relative quantitative analysis.

### Biochemical analysis

Plasma samples (50 µL, n = 10 for each group) were assayed for the absolute content of selected low molecular mass metabolites (glucose, urea, lactate, glycerol) using commercial assay kits (Nanjing Jiancheng Ltd. Co., Nanjing, China). In addition, plasma osmolality was measured by a Micro-Osmometer (OM806; Loser) using appropriate NaCl standards.

### Statistical analysis

The data from biochemical analysis of glucose, urea, lactate, and glycerol content were examined for normality of distribution and homogeneity of variance, and then were analyzed by using a Student’s t-test using IBM SPSS 20.0 software (SPSS Inc., Chicago, USA). Data are shown as means ± s.e.m. and a significance level of 0.05 (*P* < 0.05) was accepted.

Multivariate statistical analysis including principal component analysis (PCA) and orthogonal projections to latent structures discriminant analysis (OPLS-DA) along with Student’s t-test were used to analyze the metabolomics data by SIMCA (V15.0.2, Sartorius Stedim Data Analytics AB, Umea, Sweden). R^2^X, R^2^Y, and Q^2^ parameters describe the optimization of the analytic model. Afterward, permutation tests with 200 iterations were applied to validate the OPLS-DA model. Q^2^ described the predictive ability of the derived model. R^2^ implied the explanation capability towards original data and was used to evaluate whether the models were over-fitted. The OPLS-DA model was reliable and not over-fitted when Q^2^ (cum) and R^2^ (cum) values of Y-permuted models to the left were lower than that of original models to the right and Q^2^ (cum) regression lines have a negative intercept. Significant differences in metabolites between summer and winter were screened based on thresholds with *P* < 0.05 and the variable importance for the projection (VIP > 1) values. Pathway analysis was performed by combining quantitative enrichment analysis and topology analysis on MetaboAnalyst 3.0 (http://www.metaboanalyst.ca) online software using characterized KEGG pathways for *N. parkeri* as the back-end knowledge [45].

## Results

### LC-MS analysis of metabolites in *N. parkeri* plasma between two seasons

**Fig. 2:**
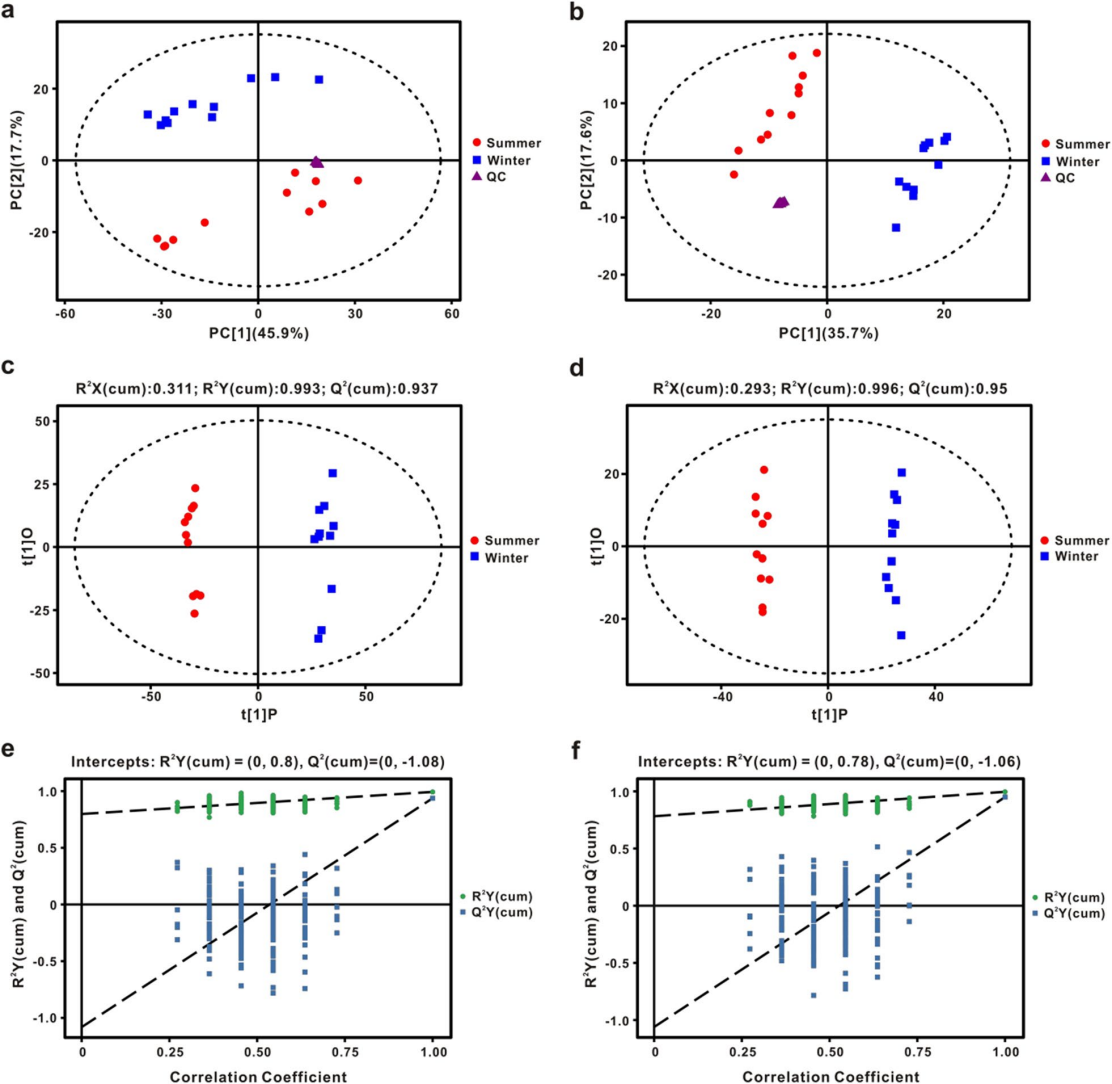
Principal component analysis (PCA) score plots, the orthogonal projection to latent structures discriminant analysis (OPLS-DA) scores plots, and permutation tests for plasma metabolites in positive ion mode (**a**, **c**, **e**) and negative ion mode (**b**, **d**, **f**). Three groups are distinguished: summer-collected frogs, blue; winter-collected frogs, red; and Quality Control (QC) samples, purple

After data preprocessing, 4598 and 2913 peaks were retained and detected in positive ion mode (POS) and negative ion mode (NEG), respectively. Using public databases (HMDB, MONA and Metlin) and the self-built database from Biotree Biotechnology Co., Ltd. (Shanghai, China) in two ion modes, 130 and 101 metabolites were identified, respectively. The PCA score plot showed that 11 QC samples were densely distributed and all were within Hotelling’s T-squared ellipse in two ion modes, suggesting a good stability of the PCA model (Fig. 2a, b). To further analyze the differences between summer and winter groups, an OPLS-DA model (Fig. 2c, d) was applied, and it showed an obvious separation between the two groups, indicating that there was a significant seasonal change in the metabolic profiles of plasma. The estimated goodness of fit of R^2^Y was 99.3% (POS) and 99.6% (NEG), respectively, and the goodness of prediction of Q^2^Y was 93.7% (POS) and 95% (NEG) which underlines the robustness of the model. Moreover, a permutation test showed that R^2^Y-intercept and Q^2^-intercept were 0.8 (POS) and − 1.08 (POS), respectively. The R^2^Y-intercept and Q^2^-intercept in NEG were 0.78 and − 1.06, respectively (Fig. 2e, f). These results indicated good accuracy and no over-fitting of the OPLS-DA model.

### Differential metabolites in the plasma between the two seasons

**Fig. 3:**
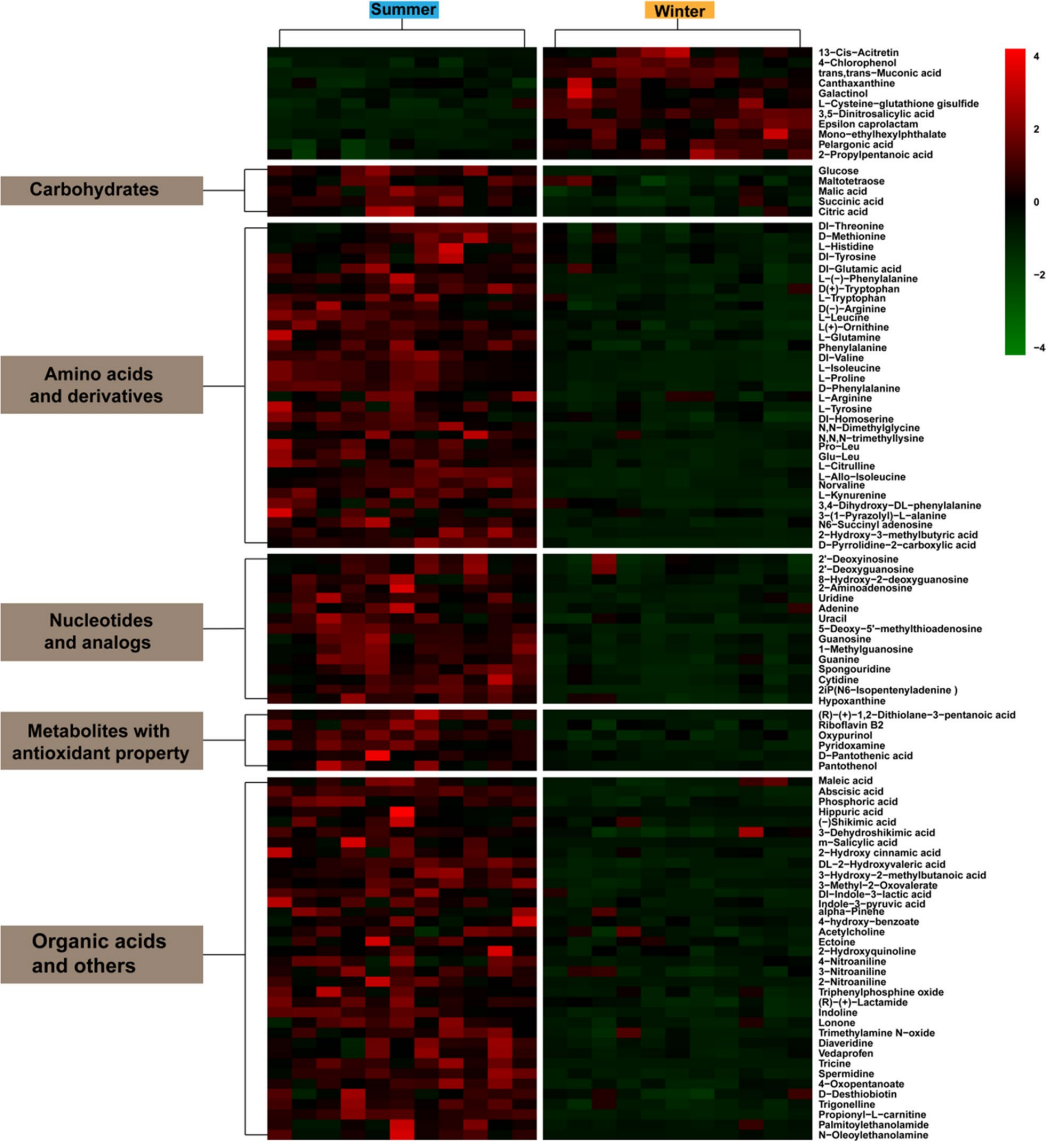
Heat map of identified differentially expressed metabolites in the plasma from summer- and winter-collected *N. parkeri*; red color indicates highly expressed metabolites, and green indicates low expressed metabolites

The LC-MS data revealed multiple metabolites that showed significant differences in abundance between summer and winter groups according to the VIP thresholds (VIP > 1) of the OPLS-DA model and the Student’s t-test (*P* < 0.05). In comparison with summer-collected frogs, there were 61 (POS) and 45 (NEG) metabolites in winter-collected frogs that changed significantly in both ion modes, respectively. In the POS ion mode, 4 qualitative metabolites increased and 57 metabolites decreased whereas 7 and 38 metabolites increased and decreased, respectively, in NEG ion mode. To further reveal the relative content of all the annotated significantly differentially expressed metabolites in the two groups and their relationships, cluster analysis was conducted, and results are presented as a heatmap (Fig. 3). Mean values and fold changes for the significantly different qualitative metabolites are shown in Additional file 1: Table S1. The number of metabolites showing decreased levels was far greater than the number showing increased values in the plasma of the overwintering frogs, as compared with summer animals.

### Metabolic pathway analysis

**Fig. 4:**
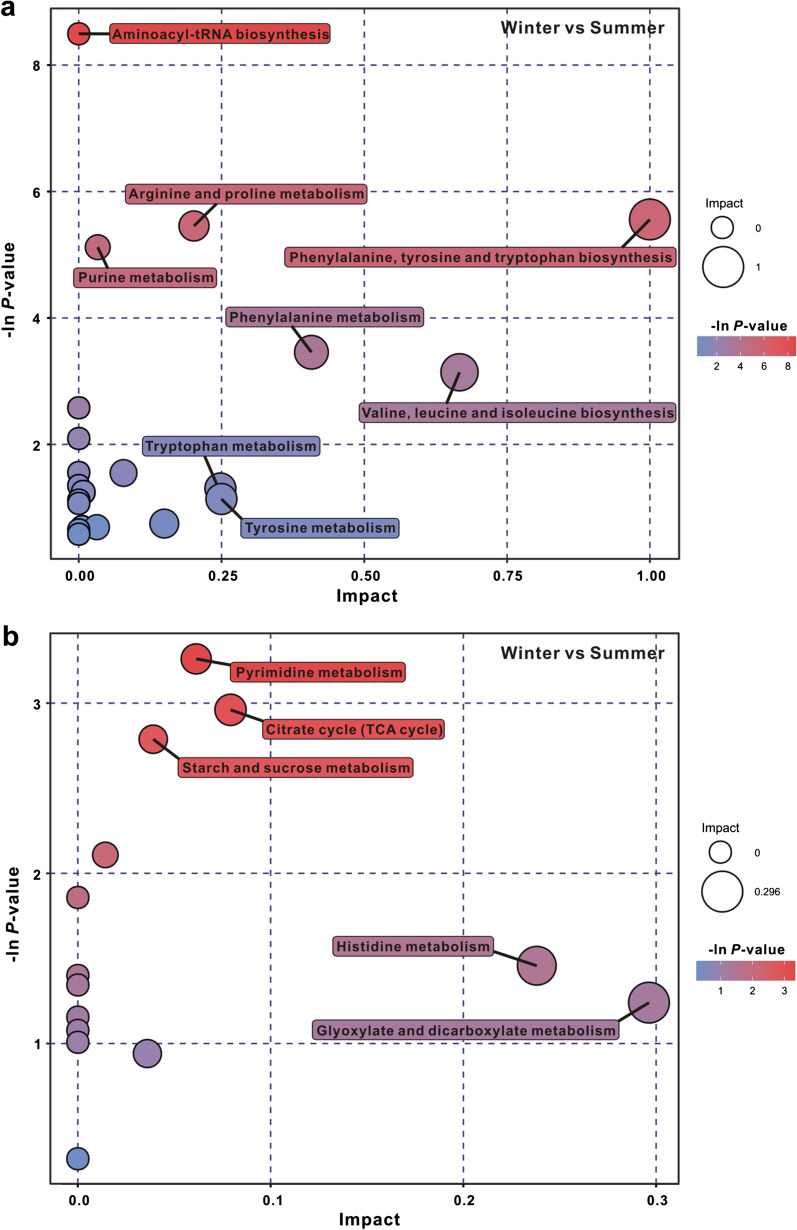
Pathway analysis of differential metabolites in plasma from summer- and winter-collected *N. parkeri* in positive ion mode (**a**) and negative ion mode (**b**). The -ln(*P* value) represents the enrichment score. The impact score (0–1) indicates the pathway topological importance of the metabolites. The color and size of each circle is based on *P* values and pathway impact values, respectively

According to pathway impact scores of > 0.05 and -ln(*P* values) of > 1.0, significant metabolic pathways were screened and visualized using a bubble chart. In POS ion mode, the metabolic pathways identified were mainly involved in amino acid metabolism including phenylalanine, tyrosine and tryptophan biosynthesis (ko00400), arginine and proline metabolism (ko00330), phenylalanine metabolism (ko00360), valine, leucine and isoleucine biosynthesis (ko00290), vitamin B6 metabolism (ko00750), tryptophan metabolism (ko00380), tyrosine metabolism (ko00350) (Fig. 4a). In NEG ion mode, pyrimidine metabolism (ko00240), citrate cycle (TCA cycle) (ko00020), histidine metabolism (ko00340), glyoxylate and dicarboxylate metabolism (ko00630) were identified as differentially expressed (Fig. 4b).

**Fig. 5:**
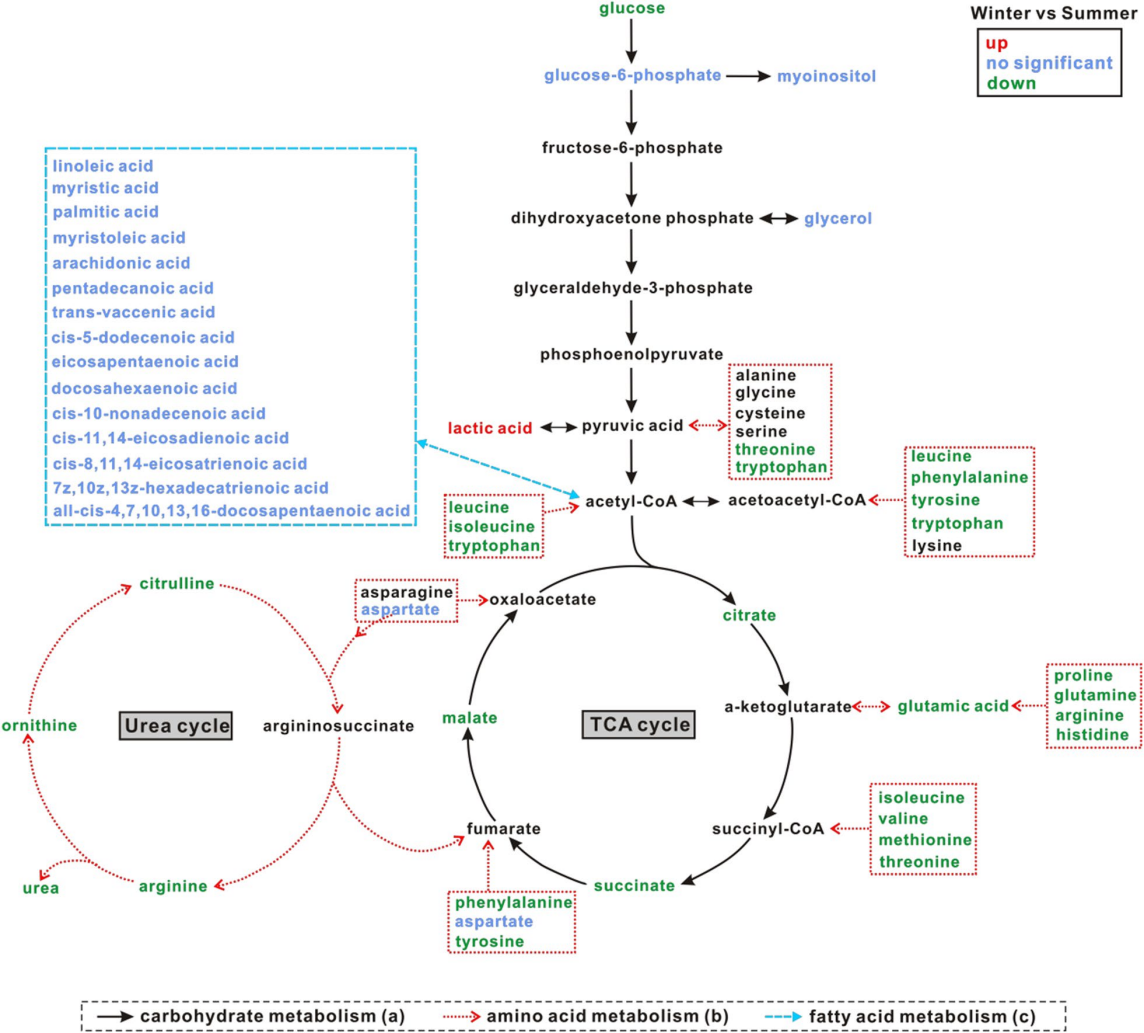
Primary metabolite changes in plasma between the two seasons. **a** Carbohydrate metabolism, **b** amino acid metabolism, **c** fatty acid metabolism. Red color indicates up-regulated metabolites, green indicates down-regulated metabolites, and blue indicates no significant change in metabolite levels

Data from LC-MS analysis showed changes in primary metabolites linked to different metabolic processes (Fig. 5). In carbohydrate metabolism, glucose, citrate, succinate, and malate all decreased significantly in plasma from winter versus summer frogs, whereas no significant changes were found in the content of glucose-6-phosphate and glycerol. Most amino acids also decreased in plasma of winter frogs, compared with summer, including threonine, tryptophan, leucine, phenylalanine, tyrosine, isoleucine, glutamic acid, proline, glutamine, arginine, histidine, valine, methionine, ornithine, and citrulline. However, multiple free fatty acids showed no significant changes in plasma during winter including cis-5-dodecenoic acid, pentadecanoic acid, trans-vaccenic acid, linoleic acid, palmitic acid, cis-11,14-eicosadienoic acid, all-cis-4,7,10,13,16-docosapentaenoic acid, myristoleic acid, cis-10-nonadecenoic acid, docosahexaenoic acid, cis-8,11,14-eicosatrienoic acid, myristic acid, 7z,10z,13z-hexadecatrienoic acid, arachidonic acid, and eicosapentaenoic acid.

### Changes in glucose, glycerol, lactate, and urea content

Compared to summer-collected frogs, the content of glucose and urea, as well as plasma osmolality decreased significantly, by 29% (*P* = 0.002), 27% (*P* = 0.027), and 27% (*P* < 0.001) in winter-collected frogs, respectively (Table 1). Lactate content showed an ~ 20% (*P* = 0.016) increase, but no significant changes were found in glycerol content in winter-collected frogs (Table 1).

**Table 1:** Changes in plasma metabolites between summer- and winter-collected *N. parkeri*

	Summer	Winter	*P*
Glucose (µmol mL^− 1^)	2.39 ± 0.15	1.69 ± 0.13**	0.002
Urea (µmol mL^− 1^)	4.19 ± 0.42	3.05 ± 0.21*	0.027
Lactate (µmol mL^− 1^)	10.63 ± 0.68	12.73 ± 0.39*	0.016
Glycerol (µmol mL^− 1^)	0.23 ± 0.02	0.23 ± 0.03	0.895
Osmolality (mOsmol kg^− 1^)	263.75 ± 8.25	192.50 ± 3.62***	0

Values are mean ± s.e.m. (n = 10). Significant differences are marked with asterisks (*P < 0.05; **P < 0.01; ***P < 0.001)

## Discussion

For many animal species, winter is a time of low metabolic activity, especially for ectotherms with metabolic rates that are largely determined by environmental temperature. Food supply is also typically interrupted so that most ectotherms rely on endogenous fuels (accumulated during summer foraging) to survive the cold months. However, regardless of their winter status, changes in biomolecules and metabolic pathways clearly support winter survival of ectotherms. The present study provides a first analysis of global biochemical changes in overwintering *N. parkeri* relative to summer-active frogs using metabonomics. These findings will contribute to elucidating metabolic and physiological adaptive mechanisms used by high-altitude amphibians to ensure winter survival for many months at temperatures close to 0 °C.

Carbohydrate metabolism is one of the first metabolic processes affected by abiotic stressors, mainly involving changes ranging from gene expression to enzyme activity [46]. When organisms need to respond to rapid increases in energy demand triggered by stress conditions, carbohydrates can respond quickly as a main fuel source, and can also be used as substrates for various biosynthesis pathways [47]. For example, in freeze-tolerant frogs, liver glycogen is not only an important fuel reserve, but it is also the carbon source for synthesizing cryoprotectants (glucose, glycerol) [8]. In the present study, glucose content showed a significant decrease in overwintering frogs, compared with summer, whether using metabonomics analysis or biochemical assay kits. Moreover, hepatic glycogen content was higher in overwintering *N. parkeri* than in summer-active frogs (unpublished data). A possible reason for decreased glucose levels is that overwintering frogs suppress their metabolic rate, reducing their energy consumption, and thereby conserve this fuel reserve to prolong their survival time [3]. This result also indicates that *N. parkeri* does not require cryoprotectants during overwintering under water, which has also been confirmed in another recent study [42]. Species that spend the winter under water do not require such cryoprotection but are vulnerable to winter-kill if their pond or lake freezes to the bottom. It is well-known that aquatic frogs that overwinter enter into a hypometabolic state by lowering ATP-utilizing processes and/or by raising the efficiency of ATP-producing pathways to extend their survival time [12, 48]. Previous studies have focused on the adaptations of the glycolytic pathway during various states of metabolic depression and also confirmed that flux through the glycolytic pathway and the tricarboxylic acid (TCA) cycle are typically depressed, such as in estivation, hibernation, or anoxia-induced hypometabolism [3, 48–50]. Citric acid is an intermediate product of the TCA cycle and is also an indicator of the energy status of cells. In this study, a lower content of citric acid suggests not only a lower flux and/or activity of the TCA cycle, but also a lower aerobic energy status. Moreover, TCA cycle pathway showed a significant change in winter as compared to summer animals. Therefore, reduced citric acid may be due to metabolic depression and a greater dependence on anaerobic glycolysis in overwintering *N. parkeri*.

Glycerol has been identified in high levels and acts as the cryoprotectant for some freeze tolerant species, such as the Siberian salamander (*Hynobius keyserlingi*) [51] and the gray treefrogs (*H. versicolor*, *H. chrysoscelis*) [8, 52]. In this study, there were no significant changes in glycerol content between the two seasons. Glycerol content (0.23 ± 0.03 µmol mL^− 1^) in *N. parkeri* plasma was similar to that found in the unfrozen *H. versicolor* (0.10 ± 0.02 µmol mL^− 1^) but far lower than the levels in the mature adult *H. versicolor* when frozen (~ 16 µmol mL^− 1^) [53]. Moreover, although hepatic glycerol content showed a significant increase in frozen *N. parkeri* (7.09 ± 0.39 µmol g^− 1^ dry tissue) [42], similar to frozen *H. versicolor* (~ 10 µmol g^− 1^) [53], it was not close to that in liver of Cope’s gray treefrog, *Dryophytes* (*Hyla*) *chrysoscelis* (up to 155 ± 27 µmol g^− 1^ dry tissue) [54]. These results indicate that glycerol was accumulated in direct response to freezing rather than as a seasonal acquisition in *N. parkeri*, as seen in *H. versicolor* [53]. However, Irwin and Lee (2003) reported that glycerol was present in high concentrations before freezing, and not further elevated by freezing in gray treefrogs *Hyla versicolor* and *H. chrysoscelis* [55]. Many variables can underlie this conflict, including geographic location, feeding conditions, sex, age, sampling time, and lab freeze/thaw conditions. However, given its very low concentration, glycerol could not act as a colligative cryoprotectant in overwintering *N. parkeri*.

Lactate as an end product of glycolysis is accumulated in plasma of overwintering *N. parkeri*. This could be supported by increased LDH activity in the liver of overwintering frogs [41]. Similar results were observed in overwintering European common lizards (*Lacerta vivipara*); lactate increased significantly in winter [56]. Our previous study also showed accumulation of high lactate levels in liver of frozen *N. parkeri*, a response to the ischemic state imposed by extracellular freezing. Elevated levels of plasma lactate may also be advantageous to overwintering *N. parkeri*, even when not frozen. High blood lactate is indicative of inter-organ transport that could redistribute this glycolytic end product to tissues and able to use it as a substrate for direct oxidation or for gluconeogenesis. Indeed, in a study of *Rana temporaria*, lactate did not accumulate in skeletal muscle but was shown to shuttle from this poorly-perfused tissue to highly-perfused tissues [57]. Furthermore, the ponds in which *N. parkeri* overwinter have low oxygen levels (4.38 ± 0.31 mg L^− 1^, n = 6) and if the pond freezes over, oxygen will be depleted over time due to the respiration of animal, plant and microbe species in the water. Hence, *N. parkeri* has likely developed a good tolerance for acidosis and lactate accumulation over evolutionary time.

It is generally accepted that urea accumulates and functions as a cryoprotectant and/or osmoprotectant in terrestrially hibernating and estivating frogs [6, 58, 59]. For instance, an Alaskan population of *R. sylvatica* accumulated up to 105 µmol mL^− 1^ urea in winter, which increased plasma osmolality [60]. However, in the aquatic environment of *N. parkeri*, urea actually decreased significantly in plasma, suggesting that neither cryo- or osmo-protective actions of urea were needed during aquatic overwintering and also that amino acid and/or protein catabolism was low during winter. Similarly, water snakes (*Nerodia sipedon*) significantly decreased their plasma osmolality in winter as compared to summer animals [61]. The relative abundance of trimethylamine N-oxide (TMAO; another osmo-regulator) was also reduced in overwintering *N. parkeri*, likely contributing to reduced plasma osmolality. Ectoine is another compatible solute that acts as an osmo-protectant [62] but is also a potent protectant against UV-induced cellular stress [63]. Plasma ectoine decreased significantly during the winter in *N. parkeri*, likely closely related to the reduced plasma osmolality and reduced UV stress.

The amino acid pool is a center of metabolic activity, and several amino acids may be accumulated in stress-tolerant species, being derived either by de novo synthesis or protein breakdown [27]. For instance, the total amino acid pool of blood increased 2.25-fold in freezing-exposed hatchling painted turtles (*Chrysemys picta marginata*) with glutamate, glutamine, serine, glycine, taurine, alanine, valine, phenylalanine, and lysine all increasing significantly [64]. Our previous study also found that the content of several amino acids increased significantly in frozen *N. parkeri*, including glutamate, threonine, tyrosine, methionine, and valine [42]. Whether this accumulation of amino acids represents an adaptive response to stress or merely describes a stress-induced metabolic imbalance of some metabolic pathways, remains unclear. However, the contents of most of the amino acids and derivatives in the plasma were significantly down-regulated in overwintering *N. parkeri* as compared to summer-active frogs. The main reason may be that overwintering frogs are not eating, thereby suppressing the need for interorgan transport of amino acids. Furthermore, due to metabolic rate depression, stored carbohydrates or lipids can likely meet energy needs over the winter months without the need to catabolize body proteins.

The present study using LC-MS technology also highlights significant changes in various metabolites that have not previously been analyzed as part of frog overwintering but may be interesting targets for further research in the field. Many of these are linked to antioxidant defense and metabolic depression and can complement our current knowledge that has mainly centered, to date, on the activities of antioxidant and metabolic enzymes that decrease in *N. parkeri* during overwintering [40, 41]. For example, levels of canthaxanthin and galactinol rose significantly in plasma of overwintering *N. parkeri*. These two metabolites have proven antioxidant (canthaxanthin, galactinol) and immune (canthaxanthin) functions [65–67] and may address specific winter survival needs for *N. parkeri*. A well-known marker of oxidative damage to DNA is 8-hydroxy-2′-deoxyguanosine [68] and the present study shows that 8-OHdG decreased significantly in plasma of overwintering *N. parkeri*. One reason for reduced 8-OHdG levels may be that the precursor of this substance, 2′-deoxyguanosine, also decreased significantly in overwintering frogs compared with summer-active individuals. Pyridoxamine (vitamin B_6_) is also an intriguing molecule that is involved in a wide range of metabolic, physiological, and developmental processes [69]. Reduced vitamin B_6_ content is consistent with the characteristics of metabolic depression of overwintering *N. parkeri*. In addition, mono-ethylhexylphthalate, a major metabolite of diethylhexyl phthalate (DEHP), can inhibit mitochondrial respiration [70]. Thus, the upregulation of mono-ethylhexylphthalate may be involved in the suppression of aerobic metabolism in overwintering *N. parkeri*. Overall, these data clearly show that two key components of long-term winter survival are attention to antioxidant defenses and metabolic depression.

## Conclusions

In summary, we report the first LC-MS-based metabolomic analysis of plasma from high-altitude overwintering frogs, *N. parkeri*. Our study shows that most amino acid metabolic pathways changed in winter and the majority of amino acids and their derivatives were decreased significantly. Moreover, citrate, succinate and malate, as intermediates in the TCA cycle, were also reduced during winter. However, our global analysis revealed that elevated levels of several metabolites indicate important functions in winter such as antioxidant defenses (canthaxanthin, galactinol) or metabolic inhibition (mono-ethylhexylphthalate). These results open new avenues for research to further explore the molecular basis of the overwintering phenotype. Overall, a thorough understanding of winter biology is important for elucidating the adaptive mechanisms used by high-altitude species to endure in their extreme environment and predicting their responses to environmental change.

## Supplementary Information

**Additional file 1: Table S1.** Significantly different qualitative metabolites in the plasma from summer- and winter-collected *N. parkeri*.

## Data Availability

The datasets used or analysed during the current study are available from the corresponding author on reasonable request.
