# Supplementary material for: Metabolic responses of plasma to extreme environments in overwintering Tibetan frogs Nanorana parkeri: a metabolome integrated analysis
Source: Front Zool. 2021 Aug 28;18:41. doi: 10.1186/s12983-021-00428-7 (PMC8403389; doi:10.1186/s12983-021-00428-7)
Supplement: Supplementary file 1 — Additional file 1: Table S1. Significantly different qualitative metabolites in the plasma from summer- and winter-collected N. parkeri. [file 12983_2021_428_MOESM1_ESM.docx]

**Supplementary Table 1.** Significantly different qualitative metabolites in the plasma from summer- and winter-collected *N. parkeri*.

| Metabolites | RT(min) | m/z | Mean summer | Mean winter | VIP | P-value | Fold change | Ion mode | Status |
| --- | --- | --- | --- | --- | --- | --- | --- | --- | --- |
| 3,5-Dinitrosalicylic acid | 36.24771 | 226.99596 | 0.059540892 | 0.489572292 | 1.808247665 | 4.27193E-07 | 8.22245474 | NEG | Up |
| 13-Cis-Acitretin | 388.33699 | 325.18368 | 0.112688145 | 0.75730432 | 1.729955669 | 0.008466031 | 6.720354846 | NEG | Up |
| Galactinol | 34.0716 | 365.10349 | 0.078628281 | 0.282827374 | 1.618353123 | 0.003826106 | 3.597018393 | POS | Up |
| L-Cysteine-glutathione gisulfide | 35.40501 | 427.09299 | 0.018057319 | 0.043137438 | 1.672143869 | 3.14323E-05 | 2.388917166 | POS | Up |
| Mono-ethylhexylphthalate | 295.16065 | 277.14389 | 0.014601897 | 0.030527108 | 1.74122441 | 0.001137056 | 2.090626212 | NEG | Up |
| Epsilon caprolactam | 176.64555 | 114.09091 | 0.080412955 | 0.158337532 | 1.822556532 | 5.19031E-05 | 1.96905501 | POS | Up |
| Canthaxanthine | 573.74928 | 565.40066 | 0.003027988 | 0.005620462 | 1.398589079 | 0.00184306 | 1.856170239 | POS | Up |
| trans,trans-Muconic acid | 31.40973 | 141.01674 | 73.0689776 | 83.8343968 | 1.703203862 | 6.0276E-05 | 1.147332282 | NEG | Up |
| 4-Chlorophenol | 31.18104 | 127.00107 | 2.647948643 | 3.015895244 | 1.588690435 | 0.00024017 | 1.138955339 | NEG | Up |
| Pelargonic acid | 285.50567 | 157.12304 | 0.289835964 | 0.318829677 | 1.752484648 | 2.42553E-06 | 1.100034904 | NEG | Up |
| 2-Propylpentanoic acid | 253.44447 | 143.1074 | 0.129189488 | 0.13661435 | 1.359260942 | 0.001419537 | 1.057472642 | NEG | Up |
| Tricine | 238.46852 | 357.15487 | 0.113689328 | 0.001252992 | 1.98598388 | 5.22367E-05 | 0.011021191 | NEG | Down |
| N-Oleoylethanolamine | 490.5324 | 326.30374 | 0.011093329 | 0.000598225 | 1.77066466 | 0.007702116 | 0.053926556 | POS | Down |
| (R)-(+)-1,2-Dithiolane-3-pentanoic acid | 33.85276 | 205.03492 | 1.063885794 | 0.120674877 | 2.011168829 | 0.000162539 | 0.113428413 | NEG | Down |
| m-Salicylic acid | 35.98979 | 139.04973 | 0.125541738 | 0.016925722 | 1.798840234 | 0.007140982 | 0.13482147 | POS | Down |
| 2iP(N6-Isopentenyladenine ) | 165.92061 | 202.10811 | 0.079912558 | 0.011064825 | 1.896316843 | 1.1038E-07 | 0.138461658 | NEG | Down |
| 1-Methylguanosine | 146.40451 | 298.11308 | 0.007032959 | 0.001038853 | 1.918997558 | 6.27911E-05 | 0.14771203 | POS | Down |
| Hippuric acid | 152.83922 | 178.05061 | 0.193911232 | 0.028918619 | 1.71960885 | 0.041443874 | 0.149133285 | NEG | Down |
| L-Kynurenine | 146.91899 | 209.09122 | 0.011258779 | 0.00183763 | 2.043128839 | 6.11565E-06 | 0.163217549 | POS | Down |
| Abscisic acid | 145.51891 | 247.12776 | 0.045660452 | 0.00770227 | 2.083819462 | 3.19834E-11 | 0.168685796 | POS | Down |
| Glu-Leu | 176.38366 | 261.14334 | 0.121664079 | 0.02121894 | 1.990891533 | 4.90215E-06 | 0.174405955 | POS | Down |
| 2-Nitroaniline | 35.95813 | 137.0354 | 0.481807621 | 0.084582256 | 1.87392194 | 0.001429844 | 0.175551926 | NEG | Down |
| Glucose | 33.1995 | 178.97236 | 0.14993175 | 0.027034288 | 1.827037319 | 2.44527E-05 | 0.180310631 | NEG | Down |
| Pantothenol | 158.13414 | 206.13789 | 0.004576405 | 0.000839901 | 1.735463979 | 0.004464196 | 0.183528462 | POS | Down |
| L-Proline | 83.54621 | 133.10473 | 0.369202054 | 0.06928911 | 2.022603058 | 9.93998E-07 | 0.187672602 | POS | Down |
| L-Isoleucine | 83.55821 | 132.10142 | 5.586521433 | 1.120431912 | 2.020643749 | 9.0762E-07 | 0.200559852 | POS | Down |
| D-Pyrrolidine-2-carboxylic acid | 35.42532 | 114.05583 | 0.668233887 | 0.136917846 | 2.054580242 | 1.51823E-07 | 0.204895095 | NEG | Down |
| Propionyl-L-carnitine | 128.23064 | 218.1378 | 0.221415116 | 0.045770646 | 2.075549011 | 2.22094E-07 | 0.206718706 | POS | Down |
| 2-Hydroxy-3-methylbutyric acid | 56.29276 | 117.05553 | 1.241780206 | 0.269676405 | 1.931818407 | 0.000132939 | 0.217169193 | NEG | Down |
| 2-Aminoadenosine | 64.02043 | 281.09846 | 0.105952399 | 0.023957826 | 1.852006049 | 0.001529092 | 0.226118766 | NEG | Down |
| L-Allo-Isoleucine | 62.09108 | 130.08708 | 20.60328353 | 4.944168138 | 1.945738488 | 1.66359E-06 | 0.239969912 | NEG | Down |
| Uridine | 89.71591 | 243.06168 | 0.35173881 | 0.091159097 | 1.748718172 | 0.000106744 | 0.259167015 | NEG | Down |
| L-Leucine | 95.28293 | 132.10141 | 15.19889801 | 3.939456389 | 1.777617575 | 0.000131807 | 0.259193554 | POS | Down |
| Dl-Valine | 52.74031 | 118.08578 | 2.304134867 | 0.600237773 | 1.96865426 | 4.87855E-06 | 0.260504618 | POS | Down |
| L-Glutamine | 31.12497 | 147.07587 | 0.449460412 | 0.117179682 | 1.894886546 | 0.000105566 | 0.260711909 | POS | Down |
| N,N-Dimethylglycine | 34.10113 | 104.07015 | 0.177119579 | 0.04679404 | 2.036438959 | 1.02056E-05 | 0.26419462 | POS | Down |
| DL-2-Hydroxyvaleric acid | 36.26058 | 117.07485 | 0.210443863 | 0.055746027 | 1.968379203 | 3.03721E-07 | 0.264897374 | NEG | Down |
| Pyridoxamine | 27.63869 | 169.09411 | 0.055951965 | 0.01502724 | 1.895603868 | 1.7377E-05 | 0.268573946 | POS | Down |
| Palmitoylethanolamide | 480.59164 | 300.28823 | 0.0041523 | 0.001120017 | 1.153869663 | 0.032338481 | 0.269734132 | POS | Down |
| 4-Nitroaniline | 58.95822 | 137.03536 | 1.882036843 | 0.513421466 | 1.004096094 | 0.00760274 | 0.272800965 | NEG | Down |
| Norvaline | 36.24016 | 116.0715 | 4.807526651 | 1.336061652 | 2.008506642 | 9.2945E-08 | 0.277910399 | NEG | Down |
| Spermidine | 26.03035 | 146.16465 | 0.172949602 | 0.049434463 | 1.976143901 | 1.37726E-05 | 0.285831611 | POS | Down |
| Phenylalanine | 103.33082 | 166.07177 | 0.010592818 | 0.003034068 | 1.685364233 | 5.37941E-06 | 0.286426902 | POS | Down |
| D-Pantothenic acid | 158.37107 | 220.11704 | 0.092134358 | 0.026629892 | 1.686923515 | 0.00614615 | 0.289033238 | POS | Down |
| Spongouridine | 91.03608 | 245.07572 | 0.045343045 | 0.01318418 | 1.910296161 | 3.0478E-06 | 0.29076521 | POS | Down |
| alpha-Pinene | 149.59283 | 137.04535 | 0.029899345 | 0.00881687 | 1.334522179 | 0.020599398 | 0.294885048 | POS | Down |
| Pro-Leu | 37.01215 | 229.15371 | 0.033803953 | 0.010458745 | 1.888802233 | 0.000154627 | 0.309394143 | POS | Down |
| N,N,N-trimethyllysine | 29.25588 | 189.15901 | 0.350594043 | 0.108886046 | 1.508211515 | 0.012549774 | 0.310575859 | POS | Down |
| 5-Deoxy-5'-methylthioadenosine | 170.04095 | 298.0954 | 0.041477109 | 0.013256782 | 1.95455627 | 2.19353E-07 | 0.319616833 | POS | Down |
| Guanosine | 135.58202 | 284.09761 | 0.175321948 | 0.056171806 | 1.654525838 | 0.000520637 | 0.320392322 | POS | Down |
| Indole-3-pyruvic acid | 93.40677 | 204.06227 | 0.021206878 | 0.006942753 | 1.620435188 | 0.002019603 | 0.327382152 | POS | Down |
| 8-Hydroxy-2-deoxyguanosine | 137.85567 | 282.08384 | 0.253489202 | 0.085501769 | 1.54836385 | 0.001164883 | 0.337299454 | NEG | Down |
| (-)Shikimic acid | 170.1869 | 172.99101 | 0.409946431 | 0.14594542 | 1.317475676 | 0.027479671 | 0.356010954 | NEG | Down |
| L(+)-Ornithine | 26.85224 | 133.09667 | 0.066074484 | 0.023645133 | 1.806504028 | 3.29812E-07 | 0.357855729 | POS | Down |
| Guanine | 135.70499 | 152.05614 | 0.093032211 | 0.034224622 | 1.751995566 | 2.23957E-05 | 0.367879272 | POS | Down |
| N6-Succinyl adenosine | 157.88748 | 384.11294 | 0.002393468 | 0.000896596 | 1.856068578 | 0.000120952 | 0.374601235 | POS | Down |
| 2-Hydroxy cinnamic acid | 93.1697 | 165.05404 | 0.250784381 | 0.095144162 | 1.51252754 | 0.004632876 | 0.379386316 | POS | Down |
| Lonone | 32.412 | 193.15393 | 0.344676914 | 0.131281485 | 1.499102414 | 0.00081694 | 0.380882733 | POS | Down |
| Indoline | 149.04457 | 120.08031 | 1.086443634 | 0.414375437 | 1.911974596 | 3.25649E-06 | 0.381405371 | POS | Down |
| D-Phenylalanine | 149.04662 | 166.08564 | 4.408661535 | 1.699797129 | 1.913812291 | 2.73266E-06 | 0.385558546 | POS | Down |
| Oxypurinol | 65.92165 | 151.0258 | 0.695720855 | 0.269410822 | 1.723991291 | 3.5456E-06 | 0.387239824 | NEG | Down |
| L-Tyrosine | 93.43038 | 182.0805 | 2.307886965 | 0.89930068 | 1.516579627 | 0.004183345 | 0.389664093 | POS | Down |
| L-(-)-Phenylalanine | 138.88263 | 164.05752 | 0.013190145 | 0.00523853 | 1.857000442 | 2.42412E-05 | 0.397154846 | NEG | Down |
| Dl-Tyrosine | 59.64167 | 180.06627 | 3.014771237 | 1.207855208 | 1.47843628 | 0.004281599 | 0.400645725 | NEG | Down |
| D(-)-Arginine | 30.06284 | 175.11832 | 0.271396135 | 0.108880209 | 1.522449477 | 0.000164624 | 0.401185554 | POS | Down |
| 3-Methyl-2-Oxovalerate | 147.68236 | 129.05551 | 4.825446347 | 1.975724951 | 1.854461288 | 1.14019E-05 | 0.409438798 | NEG | Down |
| Cytidine | 56.77484 | 244.09175 | 0.071126308 | 0.029679452 | 1.653256908 | 0.000904123 | 0.417278113 | POS | Down |
| 3,4-Dihydroxy-DL-phenylalanine | 32.2159 | 198.08412 | 0.079157124 | 0.034545612 | 1.478611239 | 0.000661163 | 0.436418234 | POS | Down |
| Dl-Indole-3-lactic acid | 173.69499 | 188.06986 | 0.414665588 | 0.181127998 | 1.689123174 | 8.79265E-05 | 0.436804989 | POS | Down |
| Triphenylphosphine oxide | 147.18622 | 279.09961 | 0.006471505 | 0.002831926 | 1.568407802 | 0.002815251 | 0.43759931 | POS | Down |
| L-Tryptophan | 173.7076 | 205.0963 | 1.026867132 | 0.45039794 | 1.682555018 | 9.63781E-05 | 0.438613649 | POS | Down |
| L-Citrulline | 32.7296 | 176.10232 | 0.136733035 | 0.060172585 | 1.770509537 | 0.000191906 | 0.440073497 | POS | Down |
| Trimethylamine N-oxide | 32.73063 | 76.07534 | 0.320073448 | 0.143836144 | 1.584260307 | 0.000742699 | 0.449384804 | POS | Down |
| Adenine | 133.84086 | 134.04701 | 0.114780386 | 0.052047976 | 1.482187954 | 0.004658018 | 0.453457059 | NEG | Down |
| D(+)-Tryptophan | 163.79245 | 203.08225 | 1.575592343 | 0.716305688 | 1.693452086 | 8.40223E-05 | 0.454626281 | NEG | Down |
| Succinic acid | 27.78093 | 117.01912 | 1.345352352 | 0.625625022 | 1.650364721 | 2.52867E-05 | 0.465026892 | NEG | Down |
| 3-Hydroxy-2-Methylbutanoic acid | 37.29155 | 117.05552 | 0.575255022 | 0.272846295 | 1.904248586 | 4.97377E-05 | 0.474304934 | NEG | Down |
| D-Methionine | 45.82454 | 148.04342 | 0.209931551 | 0.108043786 | 1.516827807 | 0.001391732 | 0.514661972 | NEG | Down |
| (R)-(+)-Lactamide | 31.11425 | 90.05456 | 0.343426612 | 0.180348805 | 1.775251153 | 8.8192E-06 | 0.525145106 | POS | Down |
| L-Arginine | 35.98988 | 175.10709 | 0.071746584 | 0.038077425 | 1.141715737 | 0.014194899 | 0.530721088 | POS | Down |
| Diaveridine | 169.77172 | 261.12966 | 0.002078254 | 0.001116331 | 1.452322141 | 0.002809366 | 0.53714838 | POS | Down |
| Citric acid | 27.94938 | 191.01935 | 1.239383885 | 0.674524404 | 1.247158772 | 0.020504119 | 0.544241709 | NEG | Down |
| 2'-Deoxyguanosine | 142.39449 | 266.08896 | 0.03582447 | 0.019507515 | 1.309901108 | 0.013603129 | 0.544530466 | NEG | Down |
| Dl-Glutamic acid | 32.18977 | 148.05986 | 0.267228811 | 0.145801526 | 1.516714748 | 0.001144532 | 0.545605563 | POS | Down |
| Phosphoric acid | 26.78547 | 96.95989 | 5.391496664 | 2.953174085 | 1.812881021 | 2.6654E-05 | 0.547746622 | NEG | Down |
| 2'-Deoxyinosine | 140.79246 | 251.07805 | 0.050367521 | 0.028549628 | 1.233119404 | 0.020924788 | 0.566826151 | NEG | Down |
| Trigonelline | 58.93138 | 138.0486 | 0.173941049 | 0.098905627 | 1.779294863 | 7.52649E-06 | 0.568615788 | POS | Down |
| Dl-Homoserine | 31.66302 | 120.06505 | 0.210535693 | 0.119894412 | 1.431064908 | 0.000673474 | 0.569473093 | POS | Down |
| 4-hydroxy-benzoate | 39.08004 | 137.02416 | 0.158939304 | 0.090971294 | 1.143821184 | 0.034105816 | 0.572364997 | NEG | Down |
| Vedaprofen | 179.712 | 300.2002 | 0.010930253 | 0.006265246 | 1.497716806 | 0.002591719 | 0.573202242 | POS | Down |
| Dl-Threonine | 32.0214 | 118.05076 | 0.45220367 | 0.26131854 | 1.34713664 | 0.00141374 | 0.577877974 | NEG | Down |
| L-Histidine | 35.16719 | 154.06195 | 3.50242331 | 2.031189829 | 1.353588629 | 0.013192108 | 0.579938417 | NEG | Down |
| 4-Oxopentanoate | 64.52279 | 115.03986 | 1.00278286 | 0.592975677 | 1.747843094 | 5.01574E-05 | 0.591330088 | NEG | Down |
| Malic acid | 40.68581 | 133.01399 | 0.232373953 | 0.142552979 | 1.415162046 | 0.000448748 | 0.613463675 | NEG | Down |
| Hypoxanthine | 58.93082 | 137.04532 | 3.252412434 | 2.000779834 | 1.683469664 | 5.27747E-05 | 0.615167933 | POS | Down |
| Acetylcholine | 34.89886 | 146.117 | 0.354116865 | 0.217871254 | 1.036327339 | 0.019309564 | 0.615252409 | POS | Down |
| Maleic acid | 27.65192 | 115.00344 | 0.281782953 | 0.178355643 | 1.123730586 | 0.029524321 | 0.632953984 | NEG | Down |
| 3-Nitroaniline | 58.93251 | 139.04975 | 0.00212362 | 0.001366107 | 1.418751998 | 0.002114267 | 0.643291389 | POS | Down |
| Uracil | 57.90362 | 111.01979 | 0.346130981 | 0.226194509 | 1.456556709 | 0.000519795 | 0.653493969 | NEG | Down |
| Ectoine | 35.60143 | 143.08103 | 0.068369181 | 0.045029129 | 1.078350328 | 0.034213311 | 0.658617353 | POS | Down |
| Riboflavin B2 | 197.51495 | 377.14358 | 0.034625746 | 0.022924838 | 1.400252951 | 0.002027137 | 0.662074934 | POS | Down |
| 3-(1-Pyrazolyl)-L-alanine | 29.77807 | 156.07621 | 0.198622066 | 0.136539869 | 1.100838498 | 0.035128667 | 0.68743555 | POS | Down |
| D-Desthiobiotin | 36.73024 | 215.13815 | 0.007618315 | 0.005314793 | 1.163605985 | 0.013213102 | 0.697633681 | POS | Down |
| 3-Dehydroshikimic acid | 31.04443 | 171.02718 | 22.76909479 | 17.59155592 | 1.22581983 | 0.023107272 | 0.772606732 | NEG | Down |
| Maltotetraose | 28.17884 | 684.8399 | 0.007585447 | 0.006009958 | 1.04456205 | 0.04242906 | 0.792301002 | POS | Down |
| 2-Hydroxyquinoline | 248.62917 | 144.04513 | 0.10375264 | 0.08564146 | 1.294977002 | 0.007045312 | 0.825438858 | NEG | Down |

Note: RT: retention time; m/z: mass-to-charge ratio; VIP: Variable importance in the projection; POS: in positive ion mode; NEG: in negative ion mode
